# Crystal and solution structures of human oncoprotein Musashi‐2 N‐terminal RNA recognition motif 1

**DOI:** 10.1002/prot.25836

**Published:** 2019-10-29

**Authors:** Lan Lan, Minli Xing, Maithri Kashipathy, Justin Douglas, Philip Gao, Kevin Battaile, Robert Hanzlik, Scott Lovell, Liang Xu

**Affiliations:** ^1^ Department of Molecular Biosciences The University of Kansas Lawrence Kansas; ^2^ Bio‐NMR Core Facility, NIH COBRE in Protein Structure and Function, The University of Kansas Lawrence Kansas; ^3^ Protein Structure Laboratory, NIH COBRE in Protein Structure and Function, The University of Kansas Lawrence Kansas; ^4^ Protein Production Group, NIH COBRE in Protein Structure and Function The University of Kansas Lawrence Kansas; ^5^ IMCA‐CAT Hauptman Woodward Medical Research Institute Argonne Illinois; ^6^ Department of Medicinal Chemistry The University of Kansas Lawrence Kansas; ^7^ Department of Radiation Oncology The University of Kansas Cancer Center Kansas City Kansas

**Keywords:** RNA‐binding protein, RNA‐binding pocket, nuclear magnetic resonance, crystallography, Musashi

## Abstract

Musashi‐2 (MSI2) belongs to Musashi family of RNA binding proteins (RBP). Like Musashi‐1 (MSI1), it is overexpressed in a variety of cancers and is a promising therapeutic target. Both MSI proteins contain two N‐terminal RNA recognition motifs and play roles in posttranscriptional regulation of target mRNAs. Previously, we have identified several inhibitors of MSI1, all of which bind to MSI2 as well. In order to design MSI2‐specific inhibitors and compare the differences of binding mode of the inhibitors, we set out to solve the structure of MSI2‐RRM1, the key motif that is responsible for the binding. Here, we report the crystal structure and the first NMR solution structure of MSI2‐RRM1, and compare these to the structures of MSI1‐RBD1 and other RBPs. A high degree of structural similarity was observed between the crystal and solution NMR structures. MSI2‐RRM1 shows a highly similar overall folding topology to MSI1‐RBD1 and other RBPs. The structural information of MSI2‐RRM1 will be helpful for understanding MSI2‐RNA interaction and for guiding rational drug design of MSI2‐specific inhibitors.

## 1 INTRODUCTION

The RNA‐binding protein (RBP) Musashi‐2 (MSI2) is overexpressed in many cancers, including colorectal adenocarcinomas,^1^, ^2^, ^3^ breast,^4^, ^5^ hematologic malignancies,^6^, ^7^, ^8^, ^9^, ^10^, ^11^, ^12^ lung,^13^ glioblastoma,^14^ and pancreatic cancers.^15^, ^16^, ^17^ As such, it mediates mRNA stability and translation of proteins involved in oncogenic pathways.^18^, ^19^, ^20^ Overexpression and knockdown studies indicate that MSI2 is a promising therapeutic target for cancer.^3^, ^13^, ^16^, ^21^, ^22^

**Figure 1:**
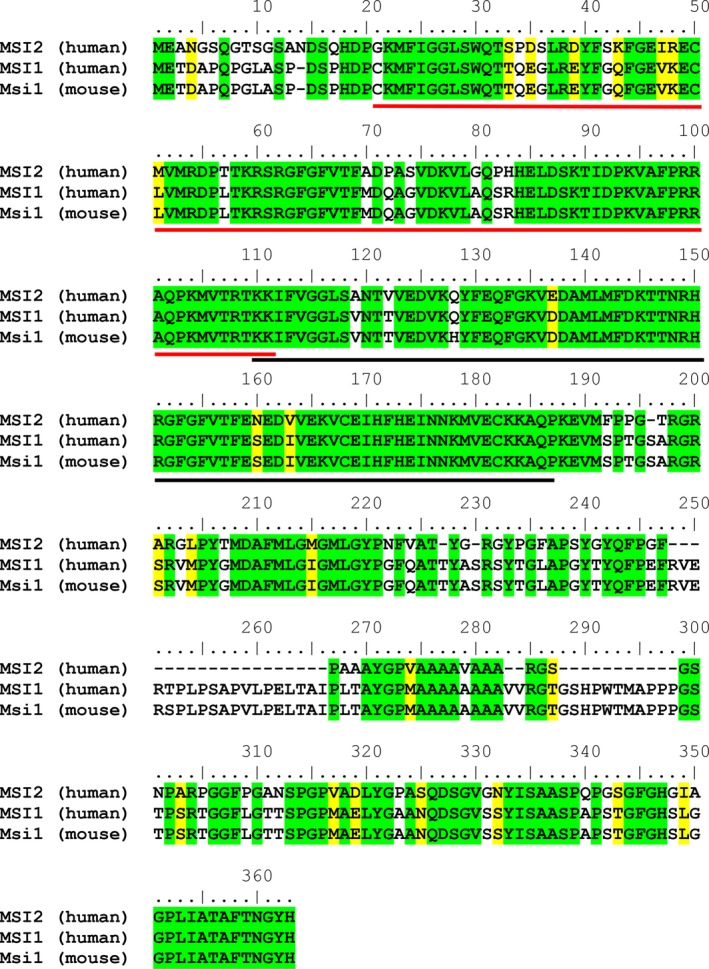
Clustal multiple sequence alignment of human MSI2, human MSI1, and mouse MSI1. Identical residues are highlighted in green, and similar residues are highlighted in yellow. The human MSI2 RRM1 and RRM2 domains are indicated by the red and black lines, respectively

Human MSI2 is a 328 amino acid protein that contains two RNA recognition motifs (RRMs) spanning G21‐K111 (RRM1) and K110‐P187 (RRM2). A BLAST^23^ search revealed that MSI2 (residues 1‐328) shares a high degree of similarity to the Musashi family proteins. Overall, MSI2 (residues 1‐328) is 69% identical to human Musashi‐1 (MSI1; residues 1‐362) and mouse MSI1 (residues 1‐362). The highest degree of similarity was observed for the N‐terminal RRMs of human MSI2 (residues 21‐187) which are 87% and 86% identical to the RNA‐binding domains (RBDs) (residues 20‐186) of human MSI1 and mouse MSI1, respectively. A multiple sequence alignment of human MSI2, human MSI1, and mouse MSI1 using Clustal Omega^24^ is shown in Figure 1. The C‐terminal region of MSI2 has no known motifs or specific function, while the N‐terminal RRMs mediate the binding to mRNAs,^25^ including those involved in the proliferation of certain cancers. As such, targeting these interactions using structure‐based drug design methods may provide a route for inhibitor development. To this end, we determined the crystal and solution NMR structures of MSI2‐RRM1 using a construct that was found to bind RNA. A high degree of structural similarity was observed between the crystal and solution NMR structures, suggesting that an orthogonal approach using both crystallographic and solution NMR methods could potentially be utilized for subsequent ligand binding studies.

## 2 MATERIALS AND METHODS

### 2.1 Fluorescence polarization assay

Fluorescence polarization (FP) assays and calculation for the Kd were carried out using our previously described methods.^26^ Three 3′ FAM‐labelled *Numb* RNAs that contain the Musashi recognition motif r(UAG) were used which included 15 nt *Numb* RNA (*Numb^15^*: 5′UAGGUAGUAGUUUUA), 8 nt *Numb* RNA (*Numb^8^*: 5′GUAGUAGU), and 5 nt *Numb* RNA (*Numb^5^*: 5′GUAGU); one 3′ FAM‐labelled 15 nt RNA with scrambled sequence was used as the control (*control^15^*). All RNAs were used at 2 nM. Fluorescence measurements were taken at room temperature using a BioTek Synergy H4 plate reader (Biotek, Winooski, VT).

### 2.2 Protein expression and purification

MSI2‐RRM1 protein used in NMR study was purified as previously described.^27^ MSI2‐RRM1 purification for crystallization was the same as the NMR except the last step of the protein was purified in the buffer of 20 mM HEPES (pH 7.5), 100 mM NaCl. To remove the His‐tag of purified MSI2‐RRM1 for crystallization, determination of the digestion efficiency of the purified proteins by TEV protease was carried out in a total volume of 50 μL containing 100 μg of protein in assay buffer (50 mM Tris‐Cl, pH 8.0, 0.5 mM EDTA, and 10 mM β‐mercaptoethanol), and various amount of TEV protease (1‐50 μg). Experiments were performed at room temperature or at 4 °C for 1 to 12 hours. The reaction was quenched by mixing with 4 × SDS‐PAGE sample buffer (40% glycerol, 564 mM Tris base, 424 mM Tris‐HCl, 8% SDS, 0.4% bromophenol blue). The degree of TEV digestion was detected by SDS‐PAGE. The quantity of the digested products was measured qualitatively by visualization on the stained gel and compared with the control experiment that was carried out without TEV in the reaction. For large‐scale reactions, purified proteins were mixed with TEV at the predetermined ratio, then dialyzed extensively against reaction buffer (50 mM Tris‐HCl, pH 8.0, 10 mM β‐mercaptoethanol, 0.5 M NaCl) and then passed through Ni column.

### 2.3 Crystallization and data collection

Purified MSI2‐RRM1 (G21‐K111) with the His‐tag removed was concentrated to 10.2 mg/mL (0.96 mM) in 150 mM NaCl, 20 mM MES pH 6.0. All crystallization experiments were setup using an NT8 drop setting robot (Formulatrix Inc.) and UVXPO MRC (Molecular Dimensions) sitting drop vapor diffusion plates at 18°C. 100 nL of protein and 100 nL crystallization solution were dispensed and equilibrated against 50 uL of the latter. Needle clusters (Figure S1) formed after 2 days and grew to their maximum size in 3 weeks from the Salt Rx screen (Hampton Research) condition E4 (2.4 M [NH_4_]_2_HPO_4_, 100 mM Tris pH 8.5). A cryoprotectant containing 80% crystallant and 20% (v/v) glycerol was layered onto the drop (2 uL), crystals were harvested and stored in liquid nitrogen. X‐ray diffraction data were collected at the Advanced Photon Source beamline 17‐ID using a Dectris Pilatus 6 M pixel array detector.

### 2.4 Crystal structure solution and refinement

Intensities were integrated using XDS^28^, ^29^ via XDSAPP^30^ and the Laue class analysis and data scaling were performed with Aimless^31^ which suggested that the highest probability Laue class was 2/*m* and space group *P*2 or *P*2_1_. The Matthews coefficient^32^ indicated that the asymmetric unit most likely contained a single molecule with (Vm = 3.3 Å^3^/Da, 62% solvent). Structure solution was conducted by molecular replacement with Phaser^33^ using a single chain from the solution NMR structure of MSI2‐RRM1 (PDB 6C8U) as the search model. The search model included G21‐V95 as the N‐ and C‐termini regions were highly flexible. Initial molecular replacement searches for a single molecule in the asymmetric unit yielded a log likelihood gain (LLG) of 18 and translation function Z‐score (TFZ) of 3 suggesting that a solution was not obtained. Although the Matthews coefficient indicated that the crystals most likely contained a single molecule in the asymmetric unit, the possibility of two molecules was also probable (2%) with Vm = 1.6 and 25% solvent. Molecular replacement search for two molecules yielded an LLG and TFZ of 42 and 5.6, respectively, indicating that an improved solution was obtained but was still weak. However, the resulting electron density maps following refinement of this solution were interpretable. The model was subjected to iterative rounds of manual model building and refinement, which further improved the electron density maps. A single subunit was then used for molecular replacement with Phaser and yielded a solution (LLG = 680, TFZ = 16) with two molecules in the asymmetric unit in the space group *P*2_1_. Further refinement and manual model building were conducted with Phenix^34^ and Coot,^35^ respectively. Disordered side chains were truncated to the point for which electron density could be observed. Structure validation was conducted with Molprobity,^36^ and figures were prepared using the CCP4MG package.^37^ Polder^38^ omit maps (Fo‐Fc) were calculated with Phenix, and structure superposition was carried out using GESAMT.^39^ Crystallographic data for MSI2‐RRM1 are provided in Table 1. Coordinates and structure factors were deposited to the Worldwide Protein Databank (wwPDB) with the accession code 6NTY.

**Table 1:** Crystallographic data for MSI2‐RRM1

	MSI2‐RRM1
Data collection	
Unit‐cell parameters (Å, ^o^)	*a* = 30.80, *b* = 57.37, *c* = 41.31, *β* = 101.3
Space group	*P*2_1_
Resolution (Å)^a^	40.51‐2.10 (2.16‐2.10)
Wavelength (Å)	1.0000
Temperature (K)	100
Observed reflections	27 686
Unique reflections	8271
<I/σ(I) >^a^	6.5 (1.8)
Completeness (%)^a^	99.4 (99.3)
Multiplicity^a^	3.3 (3.2)
*R*_merge_ (%)^a^^,^ b	11.2 (80.1)
*R*_meas_ (%)^a^^,^ d	14.4 (92.3)
*R*_pim_ (%)^a^^,^ d	7.2 (53.3)
CC_1/2_ a ^,^ e	0.993 (0.559)
Refinement	
Resolution (Å)^a^	33.09‐2.10
Reflections (working/test)^a^	7814/440
*R*_factor_/*R*_free_ (%)^a^^,^ c	20.5/28.0
No. of atoms (protein/water)	1201/37
Model quality	
RMSD	
Bond lengths (Å)	0.008
Bond angles (^o^)	0.926
Mean *B*‐factor (Å^2^)	
All atoms	36.9
Protein	36.7
Water	37.5
Coordinate error (maximum likelihood) (Å)	0.23
Ramachandran plot	
Most favored (%)	98.1
Additionally allowed (%)	1.9
PDB code	6NTY

a Values in parenthesis are for the highest resolution shell.

b *R*_merge_ = ∑_*hkl*_∑_*i*_ |*I*_*i*_(*hkl*)‐ < *I*(*hkl*) > |/∑_*hkl*_∑_*i*_ *I*_*i*_(*hkl*), where *I*_*i*_(*hkl*) is the intensity measured for the *i*th reflection and < *I*(*hkl*) > is the average intensity of all reflections with indices hkl.

c *R*_factor_ = ∑_*hkl*_ ||*F*_obs_ (*hkl*)|‐|*F*_calc_ (*hkl*)||/∑_*hkl*_|*F*_obs_(*hkl*)|; *R*_free_ is calculated in an identical manner using 5% of randomly selected reflections that were not included in the refinement.

d *R*_meas_, redundancy‐independent (multiplicity‐weighted) *R*_merge_ 31, 61. *R*_pim_, precision‐indicating (multiplicity‐weighted) *R*_merge_ 62, 63.

e CC_1/2_ is the correlation coefficient of the mean intensities between two random half‐sets of data^64^, ^65^.

### 2.5 NMR spectroscopy and structure calculation

All NMR spectra for structure calculation were recorded at 25°C on a Bruker AVANCE 800 MHz NMR spectrometer equipped with a triple resonance cryoprobe. The NMR samples contained 400 μM‐1 mM of ^13^C and ^15^N labeled MSI2‐RRM1 in 20 mM MES (pH 6.0), 150 mM NaCl. Backbone resonance assignments of MSI2‐RRM1 were reported previously under BMRB code 27111.^27^ Side chain assignments were made by analyzing HCCH‐COSY^40^ and HCCH‐TOCSY^41^ (10.9 ms mixing time) spectra. Distance restraints were obtained using 3D ^15^N edited NOESY‐HSQC^42^ (120 ms mixing time) and ^13^C edited NOESY‐HSQC^42^ (120 ms mixing time) spectra. Spectra were processed by NMRPipe^43^ and analyzed by NMRViewJ.^44^ The^15^N{^1^H} heteronuclear nuclear Overhauser effect (HetNOE) spectrum was collected at 25°C on a Bruker AVIII 600 MHz NMR spectrometer equipped with a room temperature triple resonance inverse probe. The NMR sample contained 132 μM of ^15^N labeled MSI2‐RRM1 in 20 mM MES (pH 6.0), 150 mM NaCl. Data were processed using nmrPipe and visualized using nmrDraw and CCPN Analysis^45^ on the NMRBox^46^ platform. The reference and NOE experiments were collected in an interleaved manner. Each 2D was collected with 1024 × 128 complex data points with 64 scans. The interscan delay is set to 8 seconds for the reference experiment and 5 seconds for the NOE experiment. The NOE experiment used a train of 120° hard pulses with 18 ms delays for a total saturation time of 3 seconds.

Structure calculation was performed using Crystallography and NMR system (CNS)^47^ version 1.3. The CNS calculation used default parameters with modifications in the high temperature annealing stage (15 000 K), the second Cartesian cooling stage (10 000 K, 12 000 steps), the NOE energy term scale factor (200), the dihedral angle energy term scale factor (250), and time step (0.0028). A total of 100 structures were calculated, and the 10 structures with lowest energy were selected and superimposed. The qualities of the 10 structures were assessed by CNS and the Protein Data Bank (PDB) validation server. The ensemble of the 10 lowest energy structures and experimental restraints in CNS format were deposited to the PDB (PDB accession code 6C8U), and the complete resonance assignments of MSI2‐RRM1 were deposited to the Biological Magnetic Resonance Data Bank (BMRB accession code 30398).

## 3 RESULTS AND DISCUSSION

### 3.1 MSI2‐RRM1 binds to Numb RNA

**Figure 2:**
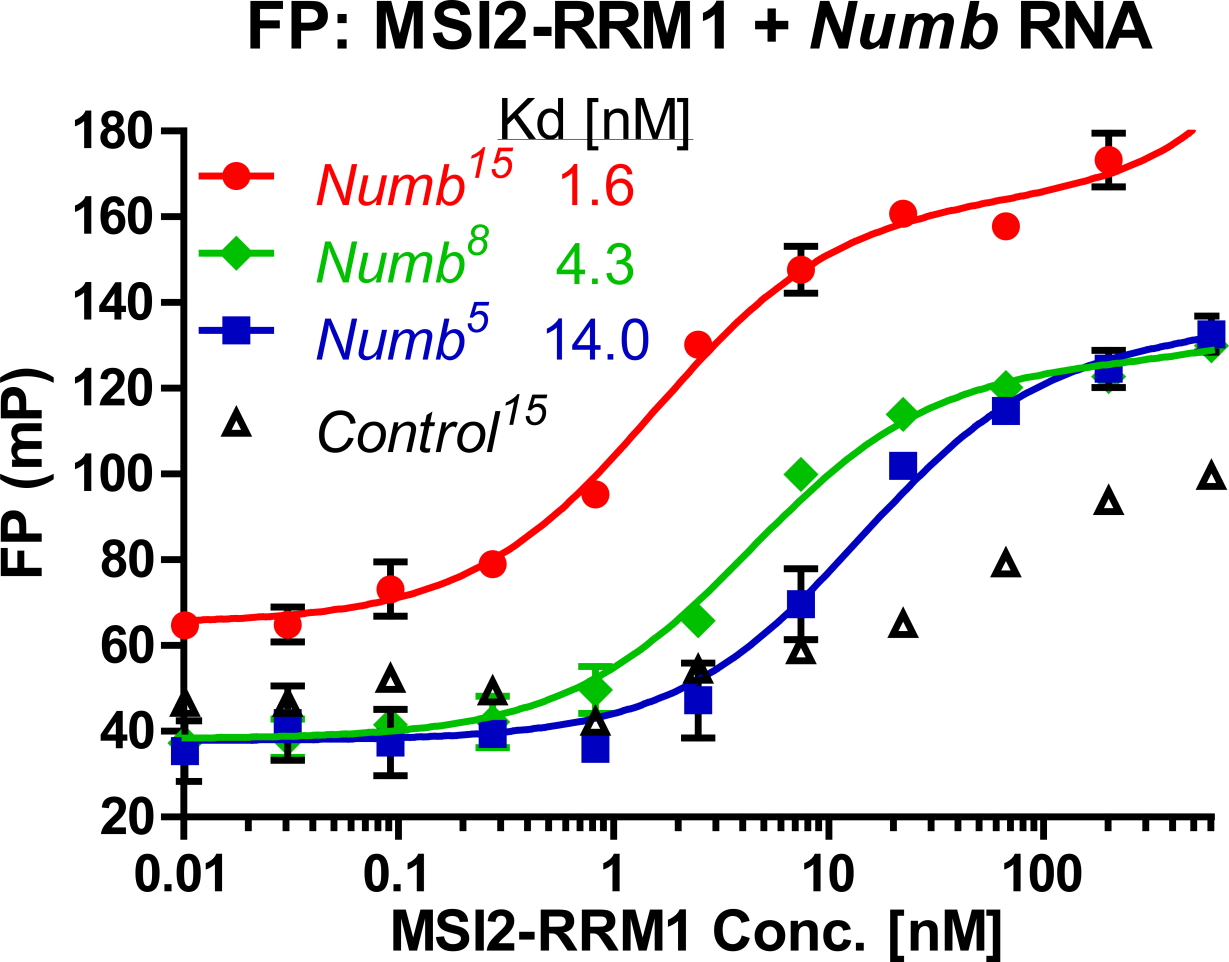
MSI2‐RRM1 binds to *Numb* RNA. Binding between RNA recognition Motif 1 (aa 21‐111) of MSI2 (MSI2‐RRM1) to three *Numb* RNAs (2 nM), but not to a *Control* RNA (2 nM) in FP assay [Color figure can be viewed at http://wileyonlinelibrary.com]

The N‐terminal RRMs (RNA‐recognition Motifs) of MSI2 mediate the binding to their target mRNAs' recognition motifs located at 3′‐UTR and one of the motifs, r(UAG), is shared between MSI1 and MSI2.^48^, ^49^ FP experiments demonstrated that all three *Numb* RNAs containing the Musashi recognition motif r(UAG) bind to MSI2‐RRM1, as indicated by the increased FP value, and MSI2‐RRM1 has a higher affinity towards *Numb* ^*15*^ compared to *Numb* ^*8*^ or *Numb* ^*5*^, as indicated by the lower Kd value (Figure 2). Importantly, compared to *Numb* ^*15*^, the 15 nt control RNA (*Control* ^*15*^) containing a scrambled sequence displayed nonspecific binding at high protein concentration (Figure 2). Importantly, these results indicate that the MSI2‐RRM1 construct adopts a functional RNA‐binding conformation in solution.

### 3.2 Crystal structure of MSI2‐RRM1

**Figure 3:**
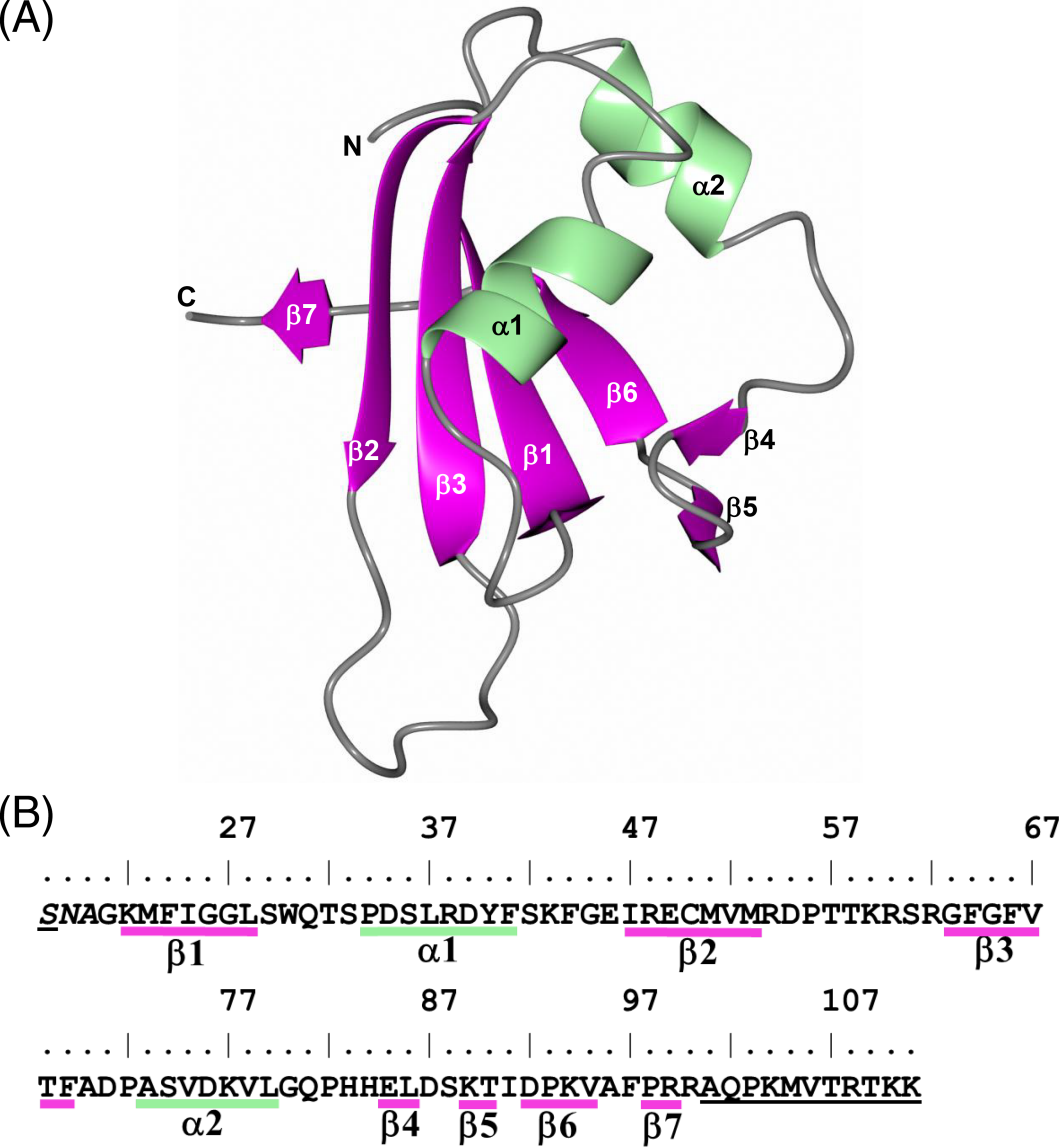
Crystal structure of the MSI2‐RRM1 subunit. A, Secondary structure elements for subunit A. B, Annotation of the secondary structure elements relative to the amino acid sequence. Residues in italics at the N‐terminus are cloning artifacts, and underlined residues were not modeled due to disorder

**Figure 4:**
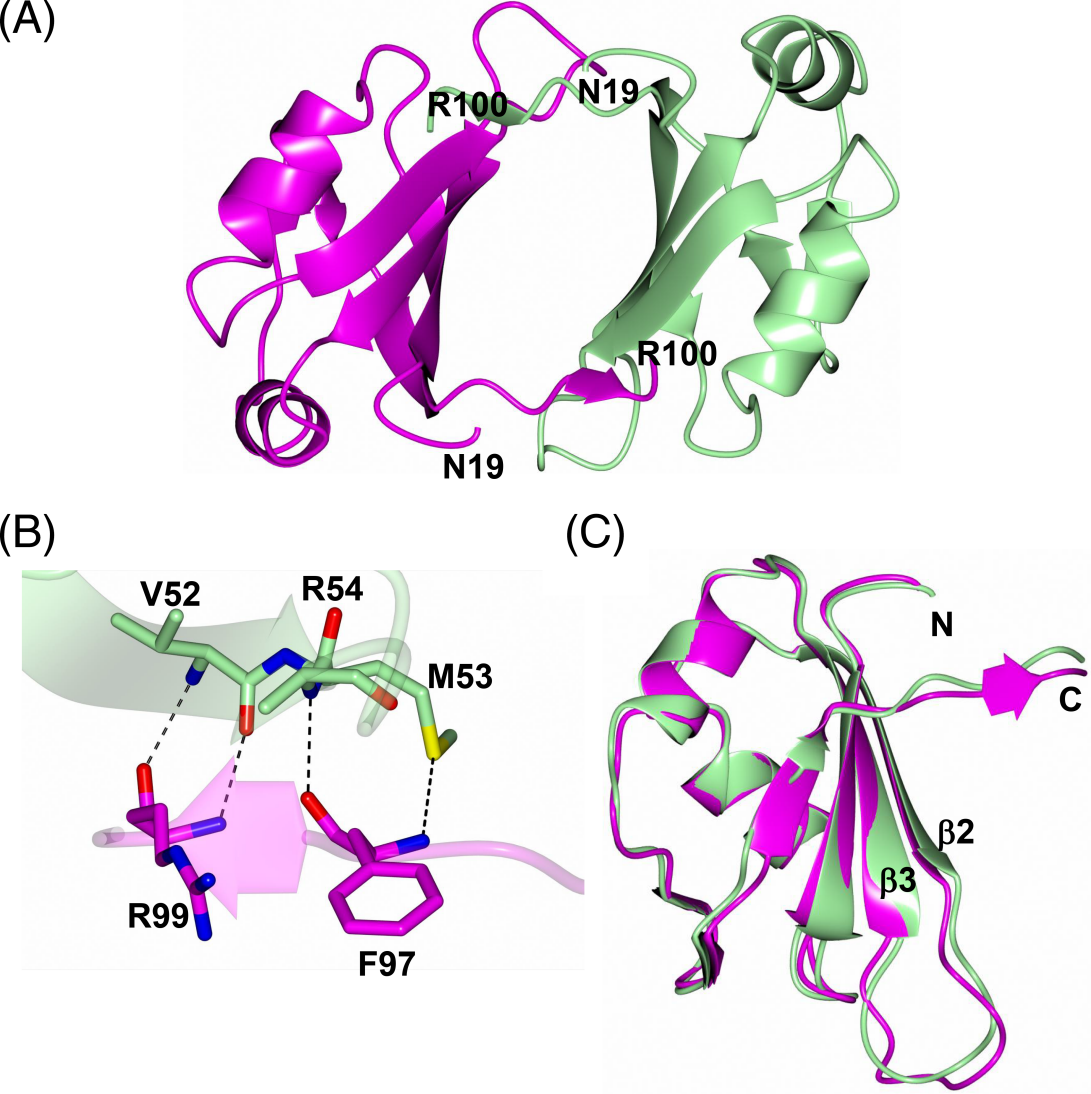
Crystal structure of MSI2‐RRM1. A) Noncrystallographic dimer with subunits A and B colored magenta and green, respectively. B) Hydrogen bond interactions (dashed lines) between the noncrystallographic dimers in the asymmetric unit. C) Superposition of subunit B (green) onto subunit A (magenta)

The crystal structure of MSI2‐RRM1 was modeled from residues G21 to R100 and consists of an antiparallel β‐strand core that is flanked by two α‐helices (Figure 3). The two subunits in the asymmetric unit form a noncrystallographic dimer in which the longest β‐stands (1, 2, 3, and 6) are positioned towards the center of the dimer (Figure 4A). The only hydrogen bond interactions between dimer subunits occurs between residues F97/R99 in the C‐terminal β7 strand of subunit A and residues V52, M53, and R54 in β2 of subunit B (Figure 4B). The structures of subunits A and B are quite similar with a root‐mean‐square‐deviation (RMSD) of 0.81 Å (80 residues aligned) between Cα‐atoms. A small difference between the subunits was in the loop that bridges β2 and β3 (Figure 4C). It should be noted, however, that the crystals are tightly packed as indicated by the Matthew's coefficient (Vm = 1.7 Å^3^/Da, 25% solvent). As such, it is possible that crystal packing could affect the conformation of the β2/β3 loop in each subunit differently. Notably, R60 in subunit A forms a contact with Q81 of a symmetry‐related molecule. However, no crystal contacts are observed with residues in the β2/β3 loop of subunit B and residues T57 and T58 were disordered.

During the review of this manuscript, a crystal structure of a MSI2‐RRM1^50^ construct spanning K22‐K104 was released. The structure is similar overall to the structure reported here and superposition of the new release and our structure yielded an RMSD of 1.11 Å between Cα atoms (78 residues). The main differences were observed in the extreme C‐terminus along with slight conformational differences in the β2/β3 loop (Figure S2).

### 3.3 NMR structure of MSI2‐RRM1

**Figure 5:**
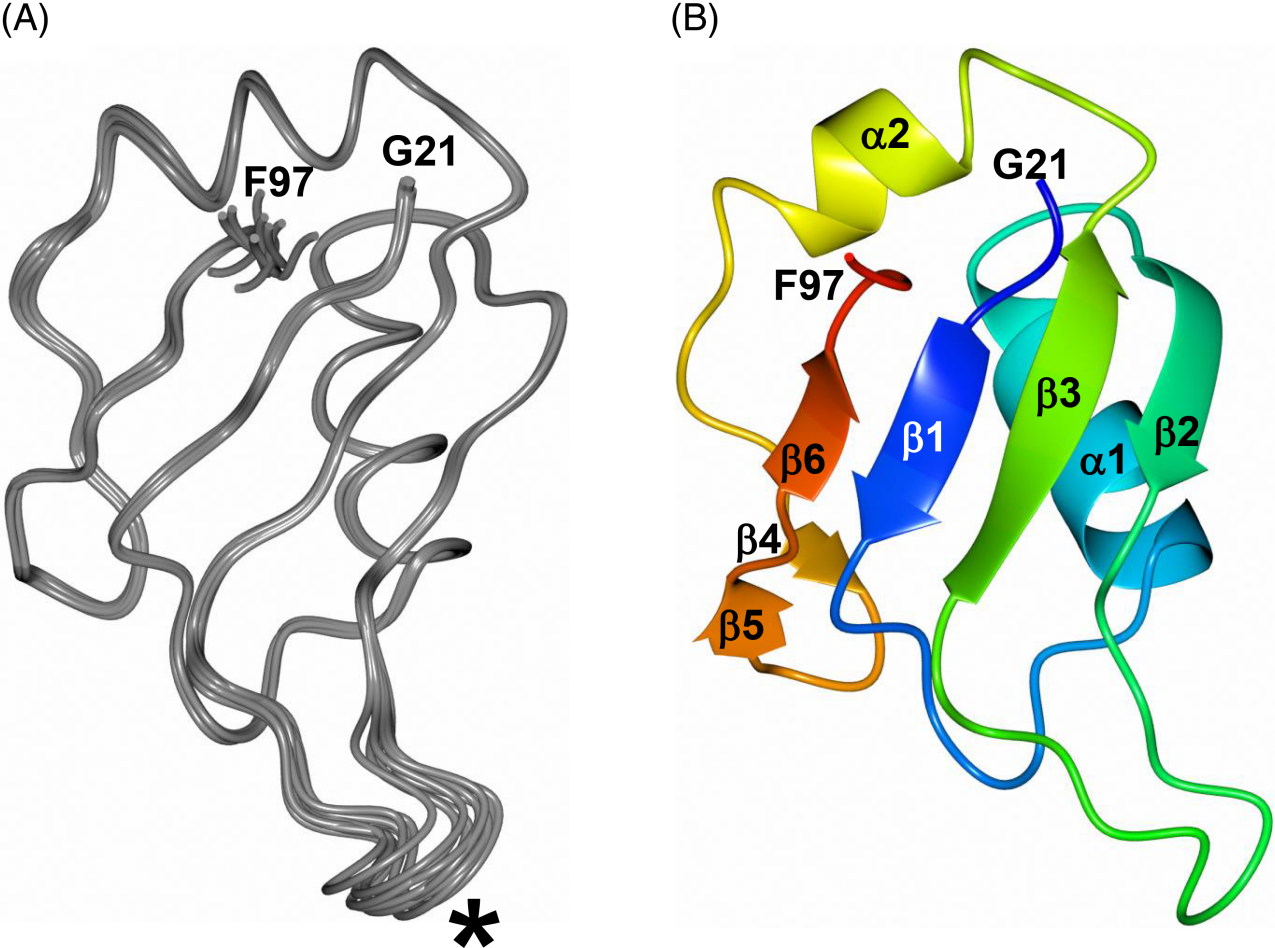
NMR solution structure of MSI2‐RRM1 with residues ‐3‐20 and 98‐111 excluded. A, Superposition of the backbone atoms for the 10 lowest energy structures of MSI2‐RRM1. The flexible loop between β2‐β3 is indicated by the asterisk. B, Ribbon diagram representation of the lowest energy structure of MSI2‐RRM1

A total of 100 structures of MSI2‐RRM1 were calculated using 1183 NOE distance restraints, 148 torsion angle restraints, 108 carbon chemical shift restraints, 100 nonglycine Hα chemical shift restraints, and 38 hydrogen bond restraints (Table 2). The superposition of the backbone atoms for the 10 lowest energy structures of MSI2‐RRM1 and ribbon diagram representation of the lowest energy structure of MSI2‐RRM1 are shown in Figure 5A,B and the statistical results of the 10 lowest energy structures are provided in Table 2. The hexahistidine tag and TEV protease cleavage site (residues M‐3‐A20) and the C‐terminus (residues P98‐K111) are not shown as they are unstructured. The well‐defined regions include residues G21‐F97, and the ill‐defined regions include residues M‐3‐A20 at the N‐terminus and residues P98‐K111 at the C‐terminus due to the lack of inter‐residue NOE's. The RMSD of the backbone atoms is 0.333 ± 0.064 Å, and the RMSD of the heavy atoms is 0.712 ± 0.086 Å. Based on Ramachandran analysis of the structural model, 98.0% of the backbone torsion angles are in the most favored conformational region, 2.0% are in the allowed region, and 0% are in disallowed regions. Similar to the crystal structure of MSI2‐RRM1, the solution NMR structure of MSI2‐RRM1 exhibits a typical ribonucleoprotein (RNP)‐type fold consisting of antiparallel β‐sheets (β1‐β6) packed against two α‐helices (α1 and α2).

**Table 2:** Statistics for the 10 lowest‐energy structures of MSI2‐RRM1

Experimental restraints	
NOE's total	1183
NOE's intra‐residue	503
NOE's sequential (|i‐j| = 1)	319
NOE's medium rang (2 ≤ |i‐j| ≤ 5)	178
NOE's long range (|i‐j|˃5)	183
Dihedral angle restraints	148
Carbon shift restraints	108
Nonglycine Hα shifts restraints	100
Hydrogen bond restraints	38
Structure results	
No. of NOE violations >0.5 Å	0
NOE violation RMSD	0.069 ± 0.000 Å
No. of phi or psi violations >5°	0
phi or psi violation RMSD	0.44 ± 0.02°
Hɑ shifts violation RMSD^a^	0.31 ± 0.01 ppm
Cɑ shifts violation RMSD^a^	1.24 ± 0.03 ppm
Cβ shifts violation RMSD^a^	0.90 ± 0.04 ppm
Bond lengths RMSD^b^	0.0077 ± 0.0001 Å
Bond angles RMSD^b^	0.77 ± 0.01°
Improper angles RMSD^b^	0.53 ± 0.01°
CNS total energy	2532 ± 7 kcal
CNS NOE energy	433 ± 5 kcal
CNS phi/psi energy	3.6 ± 0.3 kcal
wwPDB validation Ramachandran analysis	
Most favored	98.0%
Allowed	2.0%
Disallowed	0%
Backbone atoms RMSD^c^	0.333 ± 0.064 Å
Heavy atoms RMSD^c^	0.712 ± 0.086 Å
PDB code	6C8U

a Deviations from the standard chemical shifts ranges in the CNS databases.

b Deviations from the standard geometry used in CNS.

c Ill‐defined regions at residues ‐3‐20 and 97‐111 are excluded.

### 3.4 ^15^N{^1^H} HetNOE measurement

**Figure 6:**
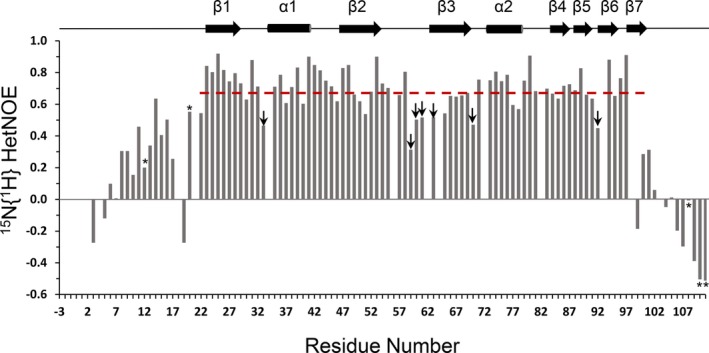
^15^N{^1^H} HetNOE values versus residue number of MSI2‐RRM1. Values marked with an asterisk are not reliable due to issues with volume measurement in either the reference or NOE spectrum. Red dashed line indicates the average HetNOE value (0.68) for residues K22‐R100. Values marked with an arrow are greater than or equal to one SD smaller than the average value for the folded core. These residues have greater flexibility than the rest of the protein, neglecting the N‐ and C‐terminal tails [Color figure can be viewed at http://wileyonlinelibrary.com]

The ^15^N{^1^H} HetNOE measured at 600 MHz for MSI2‐RRM1 is reported in Figure 6. The average HetNOE value for the folded core of MSI2‐RRM1 (residues K22‐R100) equals 0.68. HetNOE values that are one SD below the average, indicative of protein regions with fast motion on the ps‐ns timescale, were detected mostly for the N‐terminus, residue S33 at β1‐α1 loop, K59, R60, S61 at β2‐β3 loop, G63 at the beginning of β3, A70 at β3‐α2 loop, D92 at the beginning of β6, and the C‐terminal end. Overall, the HetNOE data demonstrate that the loop between β2 and β3 has greater flexibility than the rest of the protein (excluding the N‐ and C‐terminal tails) and that the loop connecting β1 and α1 is also flexible.

### 3.5 MSI2‐RRM1 crystal and NMR structure comparison

**Figure 7:**
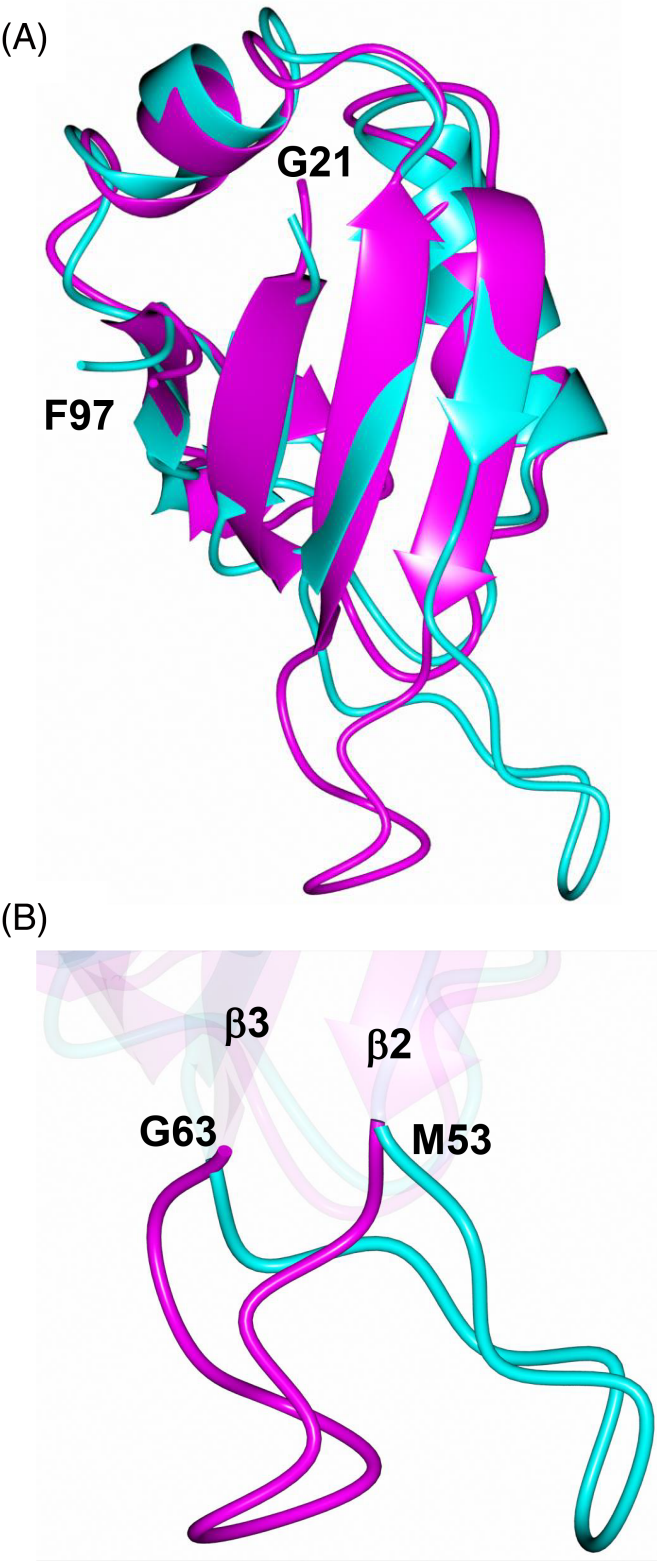
Comparison of the MSI2‐RRM1 crystal (magenta) and solution NMR (cyan) structures. A, Superposition of the NMR structure onto subunit A of the crystal structure. B, Zoomed‐in view of the conformational differences in the loop spanning β2‐β3

The crystal structure shares a high degree of similarity with the solution NMR structure of MSI2‐RRM1. Superposition of the NMR structure (model 1) onto subunit A yielded an RMSD of 1.35 Å (68 residues aligned) between Cα‐atoms (Figure 7A). Apart from the C‐terminal “tail” spanning residues A101‐K111, which could not be modeled in the crystal structure, the main difference was observed in the loop that spans β2 and β3 (Figure 7B) which is shifted approximately 8.8 Å. Additionally, the backbone HetNOE data (Figure 6) show that the β2‐β3 loop has greater flexibility than the rest of the protein, excluding the N‐ and C‐terminal tails. As noted above, the crystals of MSI2‐RRM1 are tightly packed. As such, it is possible that the symmetry‐related molecules near the β2/β3 loop could prevent this region from adopting the conformation observed in the NMR structure.

### 3.6 MSI2‐RRM1 comparison with MSI1‐RBD1 and other RBPs

The crystal structure was compared with solution NMR structures of the apo and RNA bound forms of the MSI1‐RBD1 homolog from *M. musculus*. Superposition of apo (1UAW) and RNA bound (2RS2) MSI1‐RBD1 onto chain A of the MSI2‐RRM1 crystal structure yielded RMSD of 2.17 Å (73 residues) and 0.82 Å (71 residues), respectively (Figure S3). Although the apo MSI1‐RBD1 structure displayed a higher RMSD, the overall structure is quite similar. The main differences arise in the loops connecting the helical and sheet secondary structure elements.

**Figure 8:**
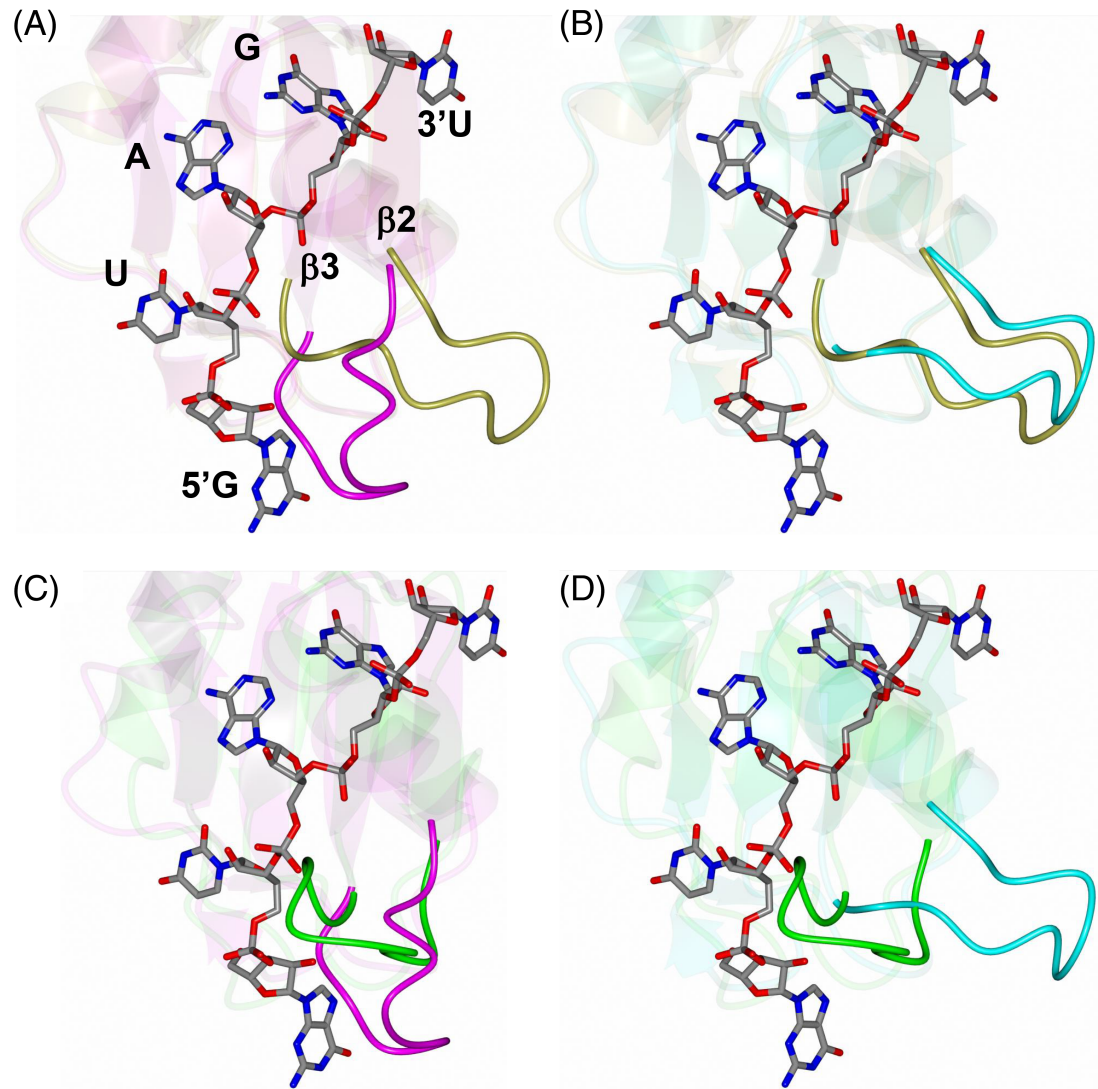
Comparison of the MSI2‐RRM1 crystal (magenta) and solution NMR (cyan) structures with RNA bound MSI1‐RBD1 (2RS2, gold) and apo MSI1‐RBD1 (1UAW, green). The RNA from 2RS2 is rendered as cylinders. The loop connecting β2‐β3 is highlighted in each panel. A, MSI2‐RRM1 crystal structure with 2RS2. B, MSI2‐RRM1 NMR structure with 2RS2. C, MSI2‐RRM1 crystal structure with 1UAW. D, MSI2‐RRM1 NMR structure with 1UAW

Evidence presented above suggests that the loop connected β2 and β3 displays a high degree of flexibility. Comparing this region of the MSI2‐RRM1 crystal structure with the lowest energy conformation of the RNA bound form of MSI1‐RBD1, revealed that this loop clashes with RNA and would need to undergo a conformational change to accommodate binding (Figure 8A). Interestingly, the MSI2‐RRM1 solution NMR structure appears to adopt a conformation similar to the RNA bound form in this region (Figure 8B). This is further supported by comparison with the lowest energy conformation of the apo structure of MSI1‐RBD1. As shown in Figure 8C,D, the β2/β3 loop in crystal structure of MSI2‐RRM1 adopts a similar conformation observed for the apo MSI1‐RBD1 structure.

**Figure 9:**
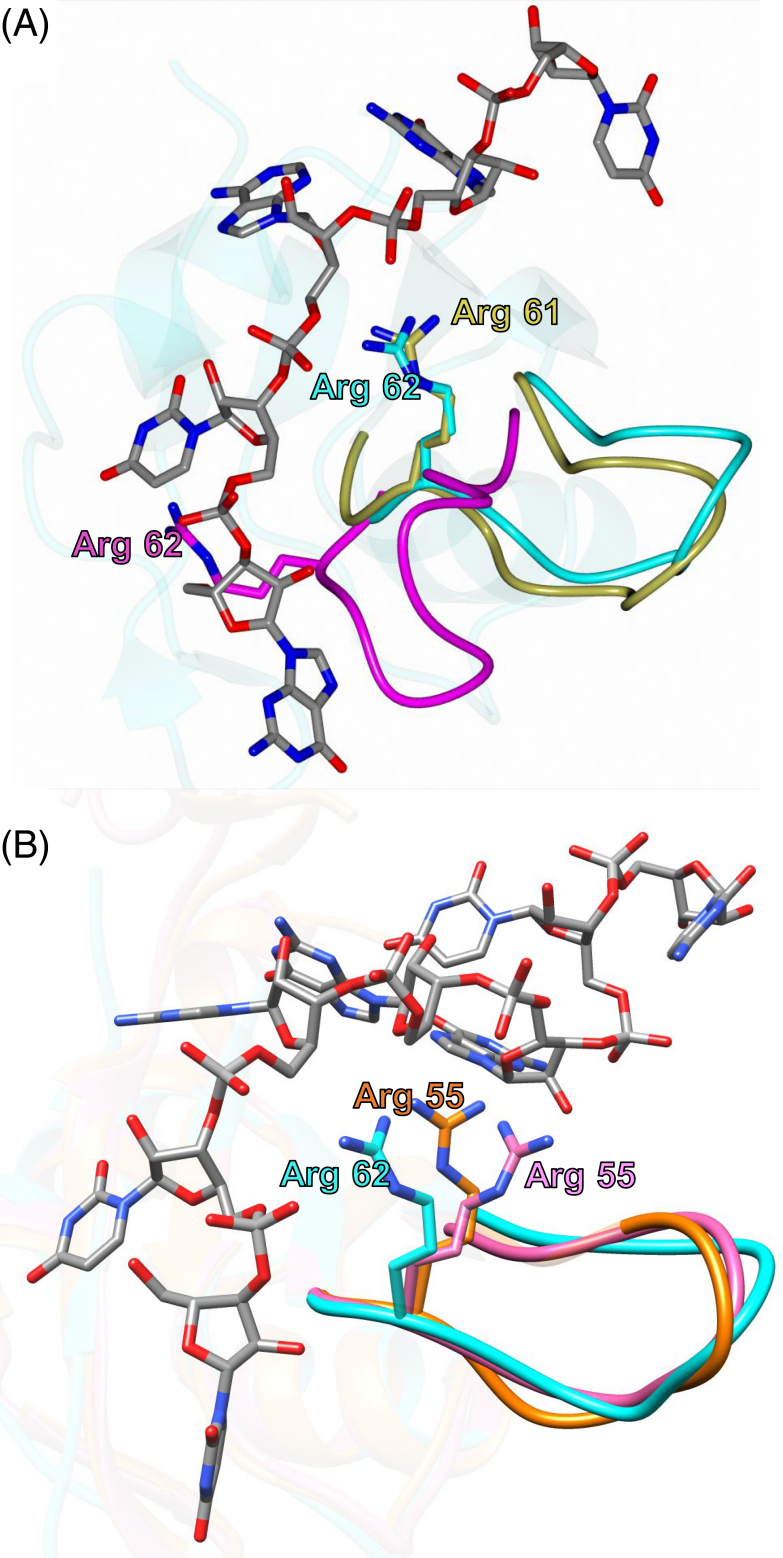
A, Comparison of the Arg 62 conformation in the MSI2‐RRM1 crystal (magenta), solution NMR (cyan) structures with Arg 61 of RNA bound MSI1‐RBD1 (2RS2, gold). B, Comparison of the Arg 62 conformation in the MSI2‐RRM1 solution NMR (cyan) structure with Arg 55 of apo HNRNP A1‐RRM1 (2LYV, orange) and RNA bound HNRNP A1‐RRM1 (5MPG, pink)

The MSI2‐RRM1 solution NMR structure ensembles were also compared with the MSI1‐RBD1 ensembles. Superposition of apo (1UAW) and RNA bound (2RS2) MSI1‐RBD1 onto chain A of the MSI2‐RRM1 solution NMR structure yielded RMSD of 1.77 Å (69 residues) and 1.26 Å (77 residues), respectively. Interestingly, the NMR model ensembles for MSI2‐RRM1 and RNA bound MSI1‐RBD1 show only minor conformational differences (Figure S4A). However, the superimposed ensembles of apo MSI1‐RBD1 displayed a high degree of difference in the β2/β3 loop (Figure S4B). The apo MSI1‐RBD1 structure adopts a wide conformational range in this loop spanning approximately 14 Å. In the RNA bound MSI1‐RBD1 structure, Arg 61 was shown to form an electrostatic interaction with an RNA phosphate group which likely serves to stabilize the β2/β3 loop. This corresponding residue in MSI2‐RRM1 (Arg 62) adopts a similar conformation in the solution NMR structure but occupies the RNA binding cleft in the crystal structure (Figure 9A).

Although it is unclear why the β2/β3 loop adopts the RNA bound conformation in the solution NMR structure of MSI2‐RRM1, this feature is not unique to MSI2‐RRM1. The NMR structure of MSI2‐RRM1 displayed a high degree of similarity in the β2/β3 loop to many other apo RRMs. For example, the NMR structure of HNRNP A1 RRM1 (PDB ID: 2LYV^51^; RMSD for 70 Cα‐atoms: 1.16 Å), the crystal structure of HNRNP A1 RRM1(PDB ID: 1UP1^52^; RMSD for 73 Cα‐atoms: 1.11 Å), the NMR structure of CYP33 RRM (PDB ID: 2KYX^53^; RMSD for 61 Cα‐atoms: 1.16 Å), the NMR structure of Hu antigen C RBD1 (PDB ID: 1D8Z^54^; RMSD for 61 Cα‐atoms: 1.47 Å), the crystal structure of hnRNP A18 RRM (PDB ID: 5TBX^55^; RMSD for 66 Cα‐atoms: 1.39 Å), and the NMR structure of drosophila sex‐lethal RBD1 (PDB ID: 2SXL^56^; RMSD for 61 Cα‐atoms: 1.61 Å). Interestingly, the NMR structures of both apo and RNA bound form of HNRNP A1 RRM1 (PDB ID 2LYV and 5MPG^57^) are similar to the NMR structure of MSI2‐RRM1 in that the β2/β3 loop and Arg 55 (corresponding to Arg 62 in MSI2‐RRM1) of the apo HNRNP A1 RRM1 adopts the RNA bound conformation (Figure 9B).

The NMR and crystal structures of MSI2‐RRM1 are similar to other RBPs as they all adopt a canonical RNP type folding: a β1α1β2β3α2β4 topology that forms a four‐stranded β‐sheet packed against two α‐helices and utilize two highly conserved regions, RNP1 and RNP2, to bind to RNA. RNP1 is located on β3 and is defined as Lys/Arg‐Gly‐Phe/Tyr‐Gly/Ala‐Phe/Tyr‐Val/Ile/Leu‐X‐Phe/Tyr, where X can be any amino acid.^58^, ^59^ RNP2 is located on β1 and is defined as (Ile/Val/Leu)‐(Phe/Tyr)‐(Ile/Val/Leu)‐X‐Asn‐Leu.^60^ The aromatic base stacking interaction between the aromatic residues in RNP1 and RNP2 and the RNA bases are a common feature in RBP/RNA complexes. Based on our previously published RNA titration work, in MSI2‐RRM1, F64 and F66 in RNP1 and F24 in RNP2 are likely to be responsible for the canonical aromatic base stacking interaction with RNA. Compared with other RBPs, MSI2‐RRM1 has a potentially unique feature in that a tryptophan in the β1‐ɑ1 loop (W30) and a phenylalanine in the C‐terminus (F97) may form noncanonical base‐stacking interactions with RNA.^27^ So far, we find that this feature is likely limited to the Musashi family proteins.

## 4 CONCLUSION

Here, we obtained the crystal and NMR structures of MSI2‐RRM1 and compared the structures to that of MSI1‐RBD1 and other RBPs. We showed that MSI2‐RRM1 adopts a canonical RNP type folding that is similar to other RBPs. The chemical shift assignments and structural information of MSI2‐RRM1 will be helpful for understanding MSI2‐RNA interaction and guiding rational drug design of MSI2‐specific inhibitors because MSI2‐RRM1 is the key motif that is responsible for the binding of MSI2 to its target mRNAs.

**Abbreviations**
MSI: Musashi
RRMs: RNA‐recognition motifs
RBDs: RNA‐binding domains
NMR: nuclear magnetic resonance
RBPs: RNA‐binding proteins
hnRNPs: heterogeneous nuclear ribonucleoproteins
RNP: ribonucleoprotien
FP: florescence polarization
PDB: protein data bank
RMSD: root‐mean‐square‐deviation
BMRB: Biological Magnetic Resonance Data Bank
CNS: Crystallography & NMR system

## CONFLICT OF INTEREST

The authors declare no competing interest.

## Supporting information


**Appendix S1**: Supporting Information
