# Supplementary material for: Crystal and solution structures of human oncoprotein Musashi‐2 N‐terminal RNA recognition motif 1
Source: Proteins. 2019 Oct 29;88(4):573–83. doi: 10.1002/prot.25836 (PMC7079100; doi:10.1002/prot.25836)
Supplement: Supplementary file 1 — Appendix S1: Supporting Information [file PROT-88-573-s001.pdf]

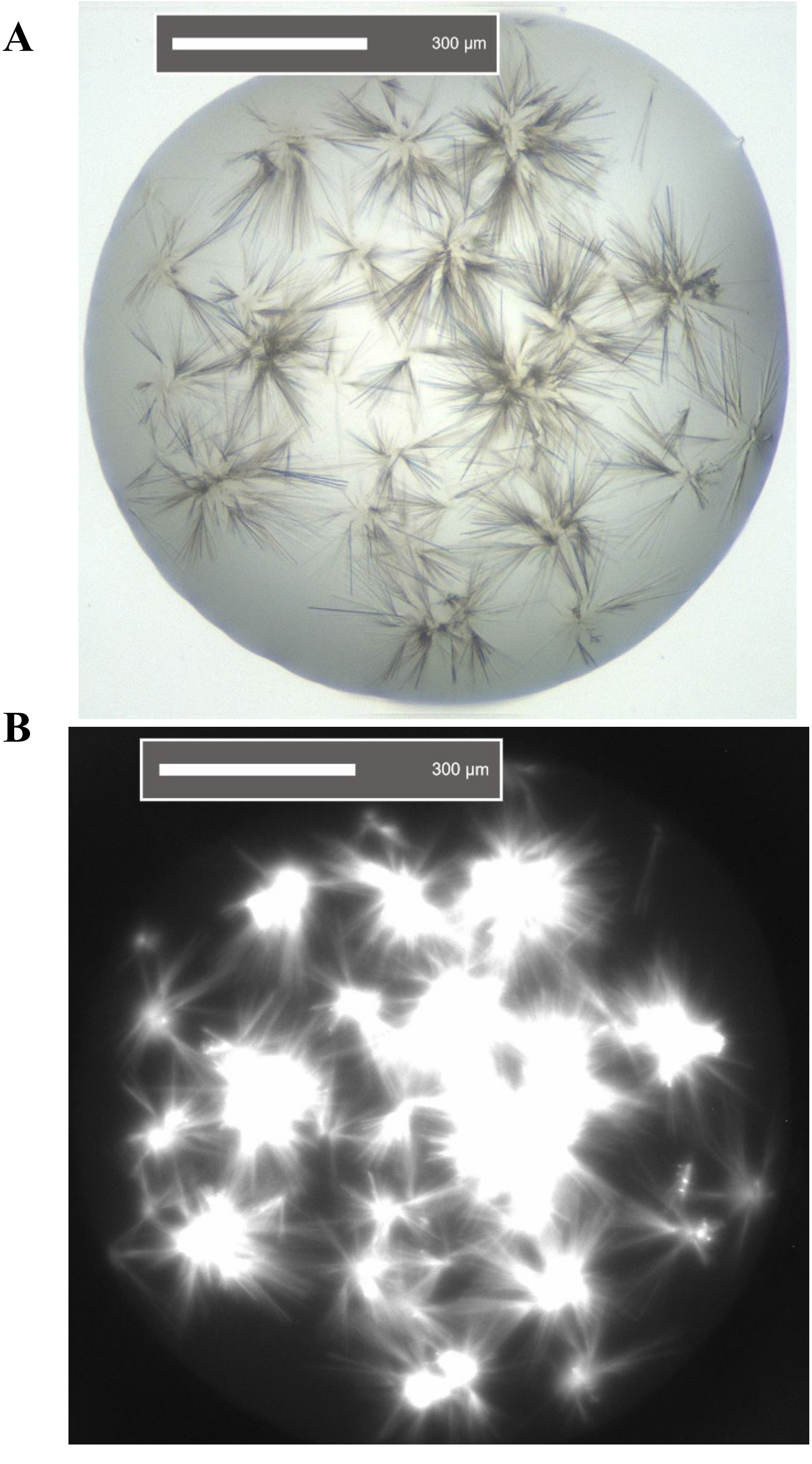

Figure S1. Crystals of MSI2-RRM1 grown from Salt Rx E4. **A**) Visible and **B**) UV images.

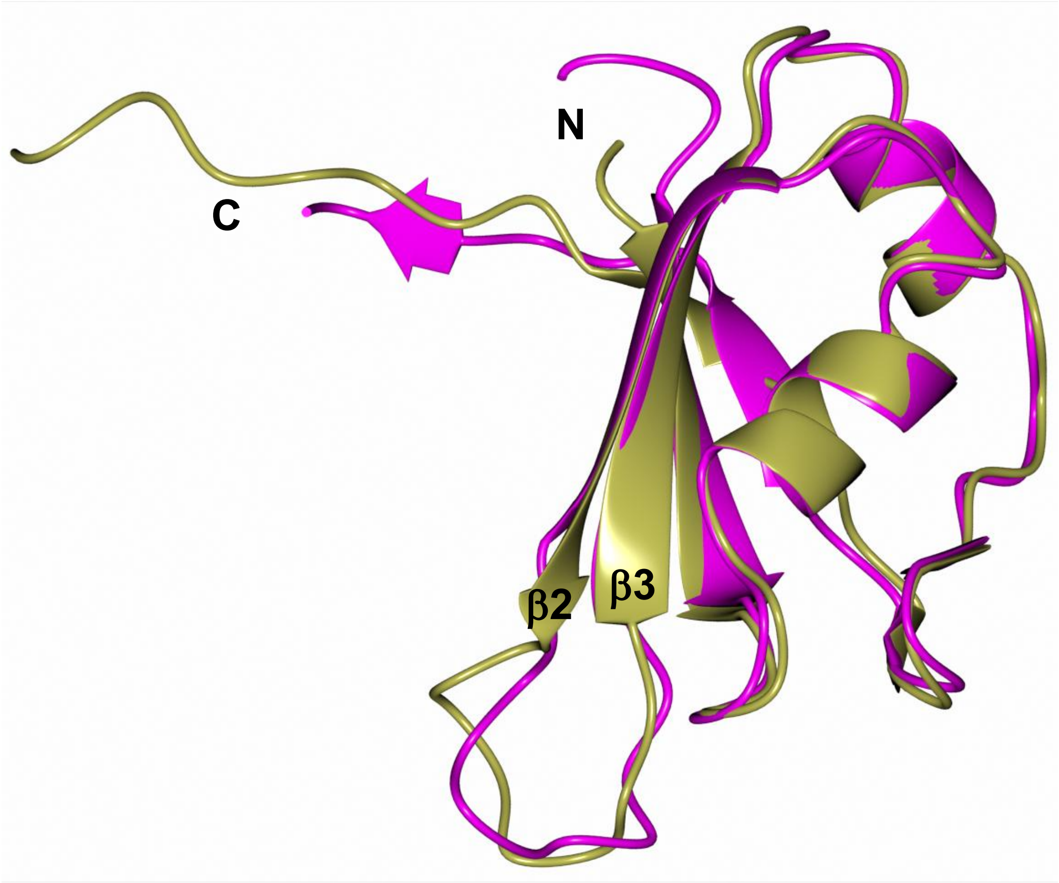

Figure S2. Superposition of MSI2-RRM1 crystal structures. The structure reported here and the recently reported structure (PDB 6DBP) are colored magenta and gold respectively.

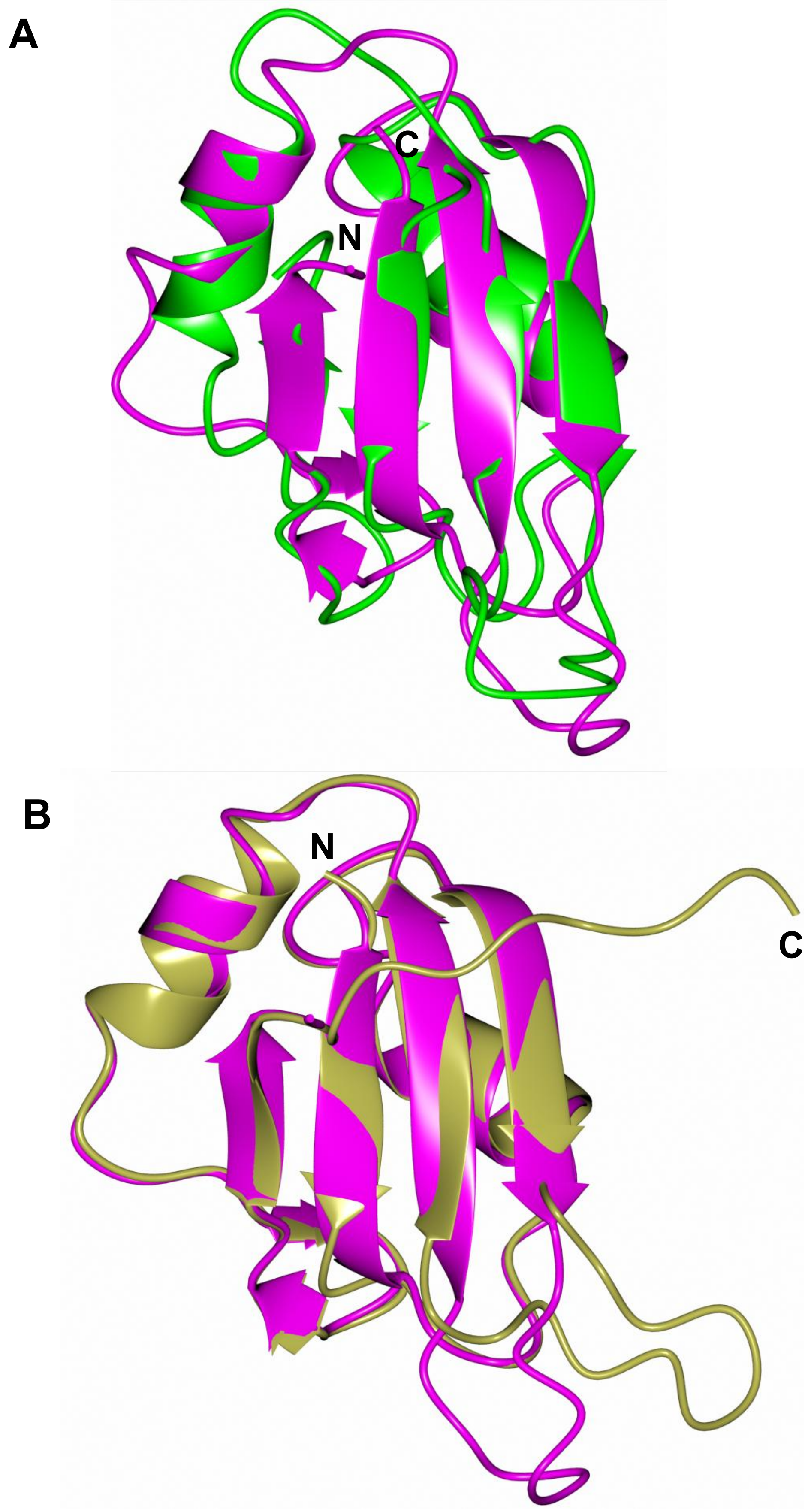

Figure S3. Superposition of MSI1-RBD1 solution NMR structures onto the crystal structure of MSI2-RRM1 (magenta). **A**) apo MSI1-RBD1 (1UAW, green) and **B**) RNA bound MSI1-RBD1(2RS2, gold).

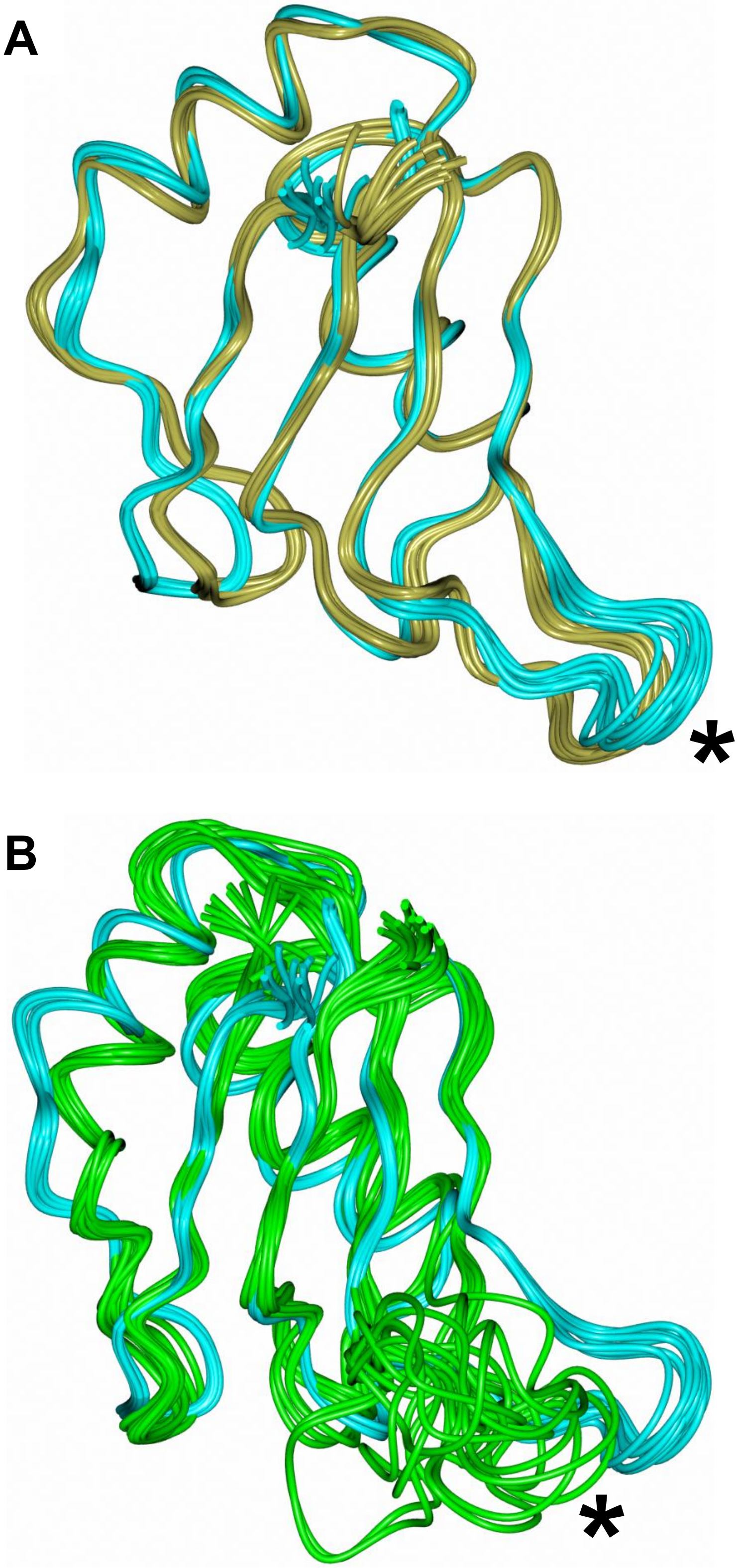

Figure S4. Superposition of NMR structure ensembles of MSI1-RBD1 and MSI2-RRM1 (cyan). **A**) RNA bound MSI1-RBD1(2RS2, gold). The RNA molecule was omitted. **B**) apo MSI1-RBD1 (1UAW, green). The flexible loop between b2-b3 is indicated by the asterisk.
